# *Pseudomonas fluorescens* DN16 Enhances Cucumber Defense Responses Against the Necrotrophic Pathogen *Botrytis cinerea* by Regulating Thermospermine Catabolism

**DOI:** 10.3389/fpls.2021.645338

**Published:** 2021-02-22

**Authors:** Lin Zhu, Nana Qian, Yujun Sun, Xiaoming Lu, Haiming Duan, Lisheng Qian

**Affiliations:** ^1^School of Life and Health Science, Anhui Science and Technology University, Bengbu, China; ^2^College of Life science, Anhui Agricultural University, Hefei, China

**Keywords:** thermospermine oxidation, defense response, *Botrytis cinerea*, rhizobacteria, polyamine

## Abstract

Plants can naturally interact with beneficial rhizobacteria to mediate defense responses against foliar pathogen infection. However, the mechanisms of rhizobacteria-mediated defense enhancement remain rarely clear. In this study, beneficial rhizobacterial strain *Pseudomonas fluorescens* DN16 greatly increased the resistance of cucumber plants against *Botrytis cinerea* infection. RNA-sequencing analyses showed that several polyamine-associated genes including a thermospermine (TSpm) synthase gene (*CsACL5*) and polyamine catabolic genes (*CsPAO1*, *CsPAO5*, and *CsCuAO1*) were notably induced by DN16. The associations of TSpm metabolic pathways with the DN16-mediated cucumber defense responses were further investigated. The inoculated plants exhibited the increased leaf TSpm levels compared with the controls. Accordantly, overexpression of *CsACL5* in cucumber plants markedly increased leaf TSpm levels and enhanced defense against *B. cinerea* infection. The functions of TSpm catabolism in the DN16-mediated defense responses of cucumber plants to *B. cinerea* were further investigated by pharmacological approaches. Upon exposure to pathogen infection, the changes of leaf TSpm levels were positively related to the enhanced activities of polyamine catabolic enzymes including polyamine oxidases (PAOs) and copper amine oxidases (CuAOs), which paralleled the transcription of several defense-related genes such as pathogenesis-related protein 1 (*CsPR1*) and defensin-like protein 1 (*CsDLP1*). However, the inhibited activities of polyamine catabolic enzymes abolished the DN16-induced cucumber defense against *B. cinerea* infection. This was in line with the impaired expression of defense-related genes in the inoculated plants challenged by *B. cinerea*. Collectively, our findings unraveled a pivotal role of TSpm catabolism in the regulation of the rhizobacteria-primed defense states by mediating the immune responses in cucumber plants after *B. cinerea* infection.

## Introduction

Plants as sessile organisms inevitably suffer from biotic and abiotic stresses. Unlike animals, plants cannot avoid these stressful factors, which seriously impact plant growth and development, and the biomass of agricultural crops. During long-term evolution, a series of highly complicated and coordinated strategies has been developed to tolerate various biotic stresses, involving activation of signaling cascades that results in a wide range of stress responses. These adaptive stress responses have well been manifested by diverse biochemical and physiological alterations, such as the biosynthesis of terpenes, pathogenesis-related proteins, and polyamines (PAs; Walters, 2003; van Loon et al., 2006; Block et al., 2019).

Polyamines including putrescine (Put), spermidine (Spd), spermine (Spm), and thermospermine (TSpm) are widely found in diverse plant species, which are important for plant physiological and metabolic processes (Shi and Chan, 2014). The Put can be synthesized *via* the catalysis of the ornithine decarboxylase and arginine decarboxylase. Subsequently, the Spd synthase promotes the conversion of Put into Spd. The Spd can be successively converted into both the Spm and TSpm by the Spm and TSpm synthase, respectively (Fuell et al., 2010; Kim et al., 2014). The TSpm exists in the whole plant kingdom, whereas the Spm is only found in diverse angiosperms (Takano et al., 2012). In *Arabidopsis*, the xylem-specific *ACL5* gene is essential for the synthesis of TSpm, which participates in the regulation of vascular development by hindering the premature death of xylem elements (Kakehi et al., 2018). The functions of TSpm have been found to be associated with the modulation of diverse processes such as cell wall patterning, cell death, and xylem cell morphology (Muñiz et al., 2008; Shinohara et al., 2019; Vuosku et al., 2019). Furthermore, high-level TSpm contributes to enhancing the tolerance of *Arabidopsis* plants to *Pseudomonas viridiflava*, whereas the *acl5* mutants deficient in the synthesis of TSpm exhibit the increased sensitivity to the pathogen attacks compared with the wild type (WT) plants (Marina et al., 2013). Accordantly, silencing of *GhACL5* reduces the levels of TSpm in cotton, thereby increasing the susceptibility to *Verticillium dahlia* (Mo et al., 2015a).

In plants, the catabolism of cellular PAs can be modulated by the PA oxidases (PAOs) and copper amine oxidases (CuAOs) under abiotic and biotic stress conditions (Cona et al., 2006; Wang et al., 2009). In many cases, biotic stress can induce the synthesis of PA synthesis and further trigger its catabolism (Angelini et al., 2010). Microbial pathogens have also been shown to induce the synthesis and oxidation of PAs in the tobacco leaf apoplast (Marina et al., 2008). Upon exposure to biotic stress, the metabolic homeostasis of PA participates in regulating defense responses in plants (Takahashi et al., 2004; Gonzalez et al., 2011). Spm can function as a key signaling molecule that provokes defense responses in both the virus‐ and *P. viridiflava*-infected plants (Gonzalez et al., 2011). However, high-level Spd can enhance the susceptibility of tomato plants to *Botrytis cinerea* by attenuating the ET-mediated signaling pathways (Nambeesan et al., 2012). PAOs can catalyze the conversion of Spd and Spm into 1,3-diaminopropane, H_2_O_2_, and the corresponding aldehyde (Rea et al., 2002). In *Arabidopsis*, several PAOs promote the back conversion of Spm to Put, accompanied by generating 3-aminopropanal and H_2_O_2_ (Moschou et al., 2008). Many studies have indicated that the oxidation of PAs can impact cell-wall maturation, lignification and reinforce cell wall, leading to the increased resistance against pathogen infection (Cona et al., 2006). Overexpression of the *PAO* genes in tobacco plants induces systemic acquired resistance and enhances the cell-wall-based defenses, conferring the increased resistance against bacterial and fungal pathogens (Moschou et al., 2009). It is increasingly evidenced that the oxidation of PAs in plants by PAOs and CuAOs is correlated with plant disease resistance (Hatmi et al., 2014, 2015). Repression of the PAO and CuAO activities in the stressed-treated grapevine leaves abolishes the activation of defense systems against *B. cinerea* infection.

It has previously been indicated that interaction of plant roots with soil microorganisms can mediate plant growth and development, and adaptive responses under adverse environments (Nadeem et al., 2014; Fahad et al., 2015). Several rhizobacteria strains establish mutualistic relationships with the hosts to benefit two parties (Zhou et al., 2019). Beneficial bacteria habituated in plant rhizosphere are frequently called as plant growth promoting rhizobacteria (PGPR), which help the host inhibit phytopathogens, promote plant growth and survive under adverse conditions (Lugtenberg and Kamilova, 2009). Many studies have reported that several PGPR strains can augment the levels of PAs in host plants by upregulating the expression of PA biosynthetic genes or direct secretion of PAs, which confers the enhanced abiotic stress tolerance in plants (Zhou et al., 2016; Sen et al., 2018). However, it remains elusive whether rhizobacteria can mediate the metabolic pathways of PAs in plants and how its metabolic alterations are correlated with host immune responses.

In this study, inoculation with *P. fluorescens* DN16 increased leaf TSpm levels in cucumber plants, which were positively associated with the transcription levels of *CsACL5*, as evidenced by the RNA-sequencing data. We further assessed the impacts of DN16 on the levels of TSpm and the activities of PA catabolic enzymes. A pharmacological approach was also applied to assess the roles of DN16-mediated TSpm catabolism in the regulation of cucumber resistance against *B. cinerea* infection.

## Materials and Methods

### Plant Materials and Microbial Culture

Seeds of the cucumber (*Cucumis sativus* L.) inbred line NK23 were sterilized with 0.1% HgCl_2_ followed by rinsing with sterile water and were then cultured in 1/2 MS medium. Seven-day-old cucumber seedlings were transferred into sterile soil and placed in a plant growth chamber at 16-h day (25°C)/8-h night (23°C) cycle.

The bacterial strain tested in this study was *P. fluorescens* DN16, which was isolated from the rhizospheric soil of 2-month-old cucumber plants. DN16 was cultured in liquid potato dextrose agar (PDA) medium at 28°C. The cultures were centrifuged at 6000 rpm for 5 min and resuspended in sterile PBS solution (0.02 M, pH 7.2). The collected cells were then adjusted to the concentration of 1 × 10^8^ CFU ml^−1^ for next experiments.

For assays of pathogen infection, the foliar pathogen *B. cinerea* was cultured on PDA agar medium at 28°C for 2 weeks. Spore suspensions of *B. cinerea* were harvested with the solution (0.05 M KH_2_PO_4_ and 6 mM glucose) and 0.01% (v/v) Tween 20, adjusting to 2 × 10^5^ spores ml^−1^ (Curvers et al., 2010).

### Generation of Transgenic Cucumber Plants

To construct *CsACL5*-overexpressing vector, cDNA sequence of *CsACL5* was amplified and ligated into the pMD18-T vector (Takara, Japan). After sequencing, the cDNA sequence of *CsACL5* was digested and introduced into the plant binary vector pCAMBIA1300. To generate RNAi-*CsACL5* vector, the cDNA fragment of *CsACL5* was amplified and inserted into the intermediated vector pHANNIBAL with the sense direction by digestion of XhoI and EcoRI and antisense direction by digestion of HindIII and XbaI, respectively. Subsequently, the pHANNIBA-*CsACL5* vector was digested by NotI and ligated into the pART27 to form the recombinant plasmid RNAi-*CsACL5*. *Agrobacterium tumefaciens* EHA105 harboring the above constructed plasmids were transformed into cucumber plants as described by Wang et al. (2014).

### Pathogen Infection Tests

Assays of *P. fluorescens* DN16-induced disease resistance were carried out, in which 7-day-old cucumber seedlings grown in 1/2 MS media were transferred into plastic pots containing sterile soils and grown for 4 weeks. Then, cell suspensions of DN16 were collected and poured into the soil (5 × 10^7^ CFU g^−1^ soil), and the control treatments were inoculated with sterile PBS solution (0.02 M, pH 7.2) for 5 days. Then, the control and inoculated plants were subjected to *in vitro* and *in vivo* pathogen infection assays (El Oirdi et al., 2011). For *in vitro* tests, 5 μl spore suspensions of *B. cinerea* were dipped on detached leaves. At 5 days post infection (dpi), lesion diameters were determined. For *in vivo* tests, the leaves were sprayed with *B. cinerea* and placed in growth chamber at 100% relative humidity. At 7 dpi, disease index was evaluated as described recently by Jing et al. (2019). Moreover, the ratio of *BcActin* to *CsActin* was calculated for determining the biomass of *B. cinerea* as reported by Hu et al. (2018).

### Histochemical Detection and Determination of Fv/Fm

*In vivo* localization of dead cells and H_2_O_2_ were detected in cucumber leaves using trypan blue and diaminobenzidine (DAB) staining, respectively (Daudi and O’Brien, 2012; Tian et al., 2018). To analyze pathogen-induced cell death, leaf tissues separated from both the pathogen-infected control and inoculated plants were incubated in the trypan blue staining solution for 45 min, followed by rinsing with distilled water five times and bleaching with 75% ethanol. To observe the production of H_2_O_2_, harvested leaves were immediately immersed in the DAB staining solution for 6 h, followed by bleaching with 75% ethanol. In addition, the values of Fv/Fm were determined by a Portable Photosynthesis System CIRAS-2.

### Analyses of RNA Sequencing and Quantitative Real-Time PCR

Five-week-old cucumber plants grown in sterile soils were inoculated with or without DN16 at the density of 5 × 10^7^ CFU g^−1^ soil for 5 days, and the control and inoculated leaves were then sprayed with spore suspension of *B. cinerea* (2 × 10^5^ spore ml^−1^). Universal RNA Extraction Kit (Takara, Japan) was applied to isolate total RNAs from the control and inoculated leaves at different time points post pathogen infection. The quality and concentration of RNA samples were spectrophotometericaly detected and the contaminated DNA was digested. To conduct RNA-Seq analyses, these RNA samples from the leaves at 0, 24, and 48 hpi were used to construct cDNA libraries, and three biological replicates were carried out. Low-quality and adapter sequences were then removed from raw data that were deposited in the NCBI SRA database (no. PRJNA686802), and were then aligned to the database.^1^ Compared with the controls, several DEGs were screened from the leaves of DN16-inoculated plants at a cutoff of FDR value <0.05 and │log_2_ ratio ≥ 1.5│, and were enriched by the GO terms and KEGG pathways (Zhang et al., 2016). Furthermore, quantitative PCR (qPCR) analyses were conducted with at least three biological replicates. qPCR reactions were performed with the SYBR Premix Ex TaqTM II kit (TaKaRa, Japan) in an ABI7500 machine. Gene expression in cucumber leaves was analyzed by qPCR and the *CsActin* gene was used as an internal control for normalizing their transcription (Yang et al., 2019). Gene expression was analyzed using the 2^−ΔΔCt^ method. The information of primers was shown in Supplementary Table S1.

### Determination of PAs

The content of PAs was quantified by HPLC as reported previously by Mo et al. (2015b). Briefly, leaf tissues (0.5 g) were ground and mixed in 10 ml of ice-cold 5% perchloric acid for 1 h, and then centrifuged at 12000 rpm for 15 min at 4°C. The supernatants (0.5 ml) were mixed with 1 ml of 2 N NaOH and 7 μl of benzoyl chloride, followed by reacting for 30 min at 37°C. After that, 1.5 ml of saturated NaCl and diethyl ether was added into the mixture and was then centrifuged for 10 min at 1000 rpm. One milliliter of organic solvent phase was collected and dissolved in 100 μl of methanol and filtered by 0.22 μm microfiltration membrane. Finally, the methanol solution was detected by HPLC as reported by Mo et al. (2015b).

### Analyses of PAO and CuAO Activities and Pharmacological Treatments

Assays of leaf PAO and CuAO activities were carried out as reported previously by Hatmi et al. (2015). Leaf tissues (0.5 g) was homogenized in 1 ml of 0.25 M phosphate buffer solution, and then centrifuged at 12000 rpm for 5 min at 4°C. The supernatants were used to detect the activities of PAOs and CuAOs as described previously by Cona et al. (2006). In addition, to inhibit the activities of PA catabolic enzymes, the leaves of whole plants were pretreated with the CuAO inhibitor aminoguanidine (AG, 5 mM) or the PAO inhibitor β-hydroxyehtylhydrazine (HEH, 10 mM). After 3 days of treatments, these plants were subjected to foliar treatment with spore suspension of *B. cinerea* (2 × 10^5^ spore ml^−1^).

### Statistical Analysis

The data were analyzed with SPSS software, and all the data were indicated as the means ± SD from three biological repeats and analyzed statistically using Duncan’s multiple range tests (*p* < 0.05).

## Results

### *P. fluorescens* DN16 Induces Cucumber Resistance Against *B. cinerea* Infection

**Figure 1:**
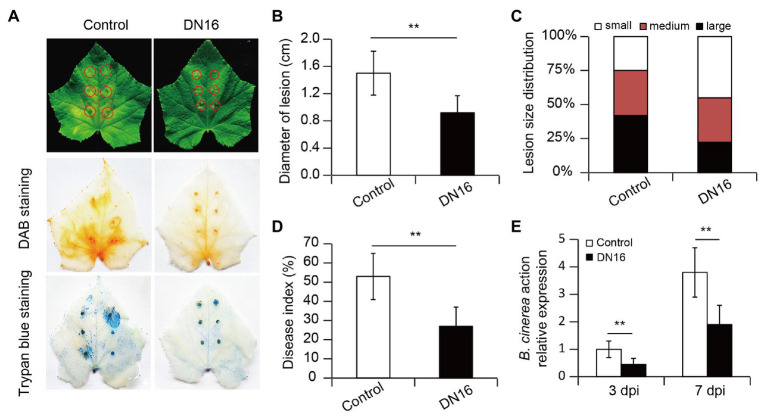
Effects of *Pseudomonas fluorescens* DN16 inoculation on the resistance of cucumber plants against *Botrytis cinerea*. Five-week-old cucumber plants were treated with or without DN16 for 5 days. Then, these plants were challenged with the foliar pathogen *B. cinerea*. **(A)** Lesion symptoms of detached leaves at 5 days post infection (dpi), and diaminobenzidine (DAB) and trypan blue staining of pathogen-infected leaves. **(B)** Lesion diameters of detached leaves. **(C)** Lesion distribution: small (LD < 5 mm), medium (5 mm < LD < 10 mm), and large (LD > 10 mm). **(D)** Both the control and inoculated leaves were sprayed with *B. cinerea* for 7 days, and disease index was calculated. **(E)** Relative expression of *BcActin* was examined at 3 and 7 dpi, respectively. The data represented the means ± SD from three biological repeats and significant differences were examined by student’s *t*-test at ^**^p < 0.01.

To examine whether *P. fluorescens* DN16-induced systemic resistance (ISR) was effective against the foliar pathogen *B. cinerea*, 5-week-old cucumber plants grown in sterile soils were treated with cell suspension of DN16 for 5 days, followed by challenging with *B. cinerea*. In the detached leaf tests, leaves of non-inoculated (control) plants exhibited larger lesion diameters and necrotic tissues at 5 dpi (Figure 1A). In contrast, leaves of DN16-inoculated plants displayed disease-resistant phenotypes, as reflected by smaller lesion diameters (Figures 1A,B). In addition, the inoculated plants had higher proportions of fewer small lesions (lesion diameter < 5 mm) than the controls (Figure 1C).

The production of H_2_O_2_ and cell death in the leaves was further examined by DAB and trypan blue staining, respectively. As shown in Figure 1A, the leaves of the inoculated plants accumulated lower H_2_O_2_ levels than those of the controls. The inoculated leaves also exhibited markedly less cell death than the control leaves. In the whole-plant inoculation assays, both the control and inoculated leaves were treated with *B. cinerea*. At 7 dpi, the inoculated plants had lower disease index than the controls (Figure 1D). Moreover, qPCR analyses showed that the inoculated leaves had lower *BcActin* transcripts than the controls at 3 and 7 dpi (Figure 1E), which was in accordance to the observed reduction of disease occurrence.

To rule out the probability that the DN16-induced disease resistance was resulted from direct impacts of DN16 on *B. cinerea*, potentially transferring of root-inoculated bacteria into leaf tissues was evaluated by culturing leaf extracts from the DN16-inoculated plants onto selective KB agar plates. However, DN16 was not detected in the inoculated leaves (data not shown). In combination with the inability of DN16 to antagonize the growth of *B. cinerea*, our data indicated that the DN16-induced systemic resistance was not attributable to microbial antagonism but rather activation of the defense systems of plant itself.

### Transcriptome Analyses of the DN16-Inoculated Plants Infected by *B. cinerea*

**Figure 2:**
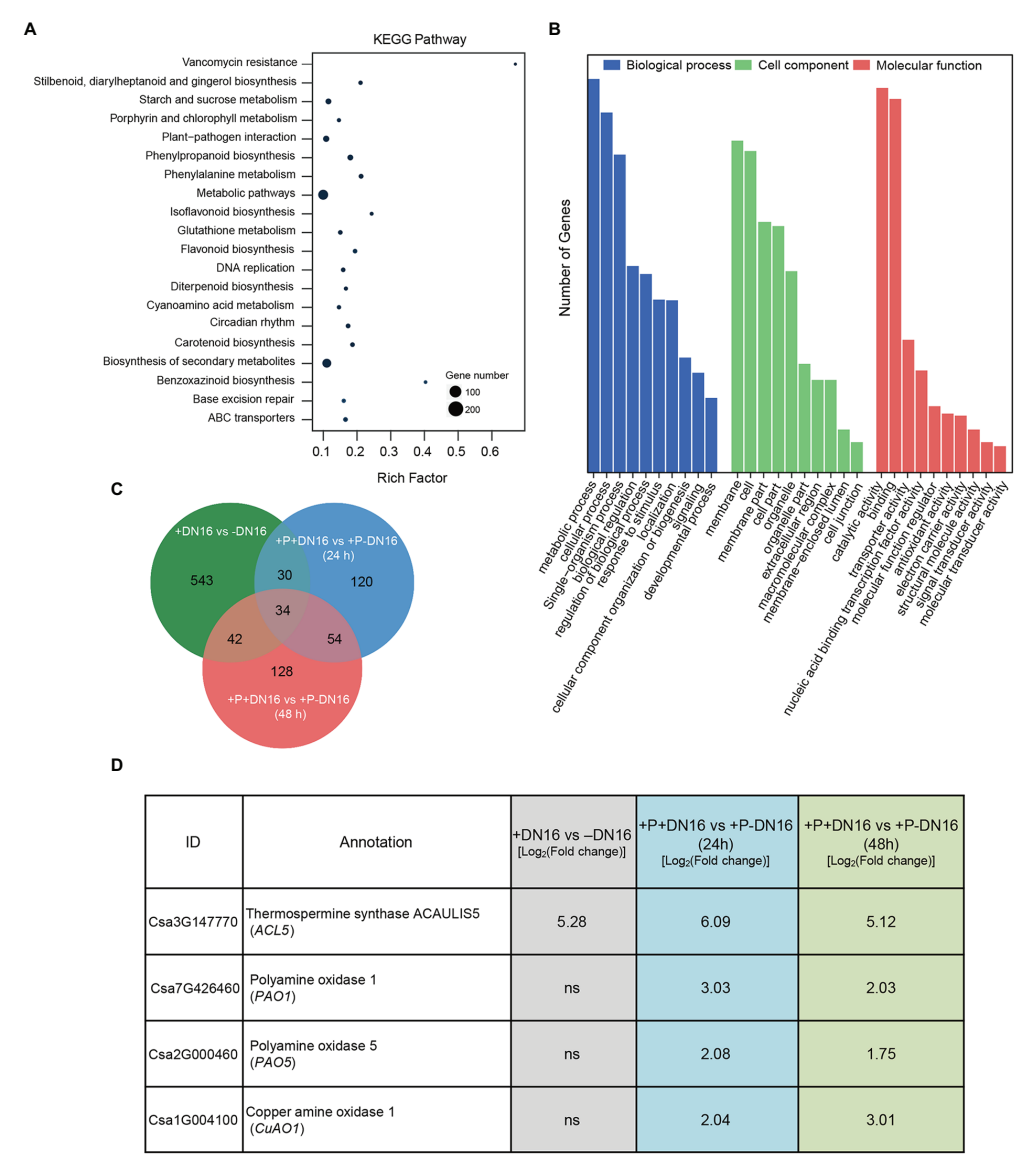
Identification of differentially expressed genes (DEGs) in cucumber plants in response to *P. fluorescens* DN16 and/or *B. cinerea*. Five-week-old cucumber plants were treated with or without DN16 for 5 days. Then, both the control and inoculated leaves were sprayed with *B. cinerea*. The leaves at 0, 24, and 48 dpi were harvested for the RNA-Seq analyses. **(A)** KEGG pathway and **(B)** GO enrichment analyses of DEGs at 0 dpi. **(C)** Venn diagrams of shared DEGs among different experimental groups. **(D)** Expression profiles of four polyamines (PA)-related genes (*CsACL5*, *CsPAO1*, *CsPAO5*, and *CsCuAO1*) in both the control and inoculated leaves challenged with or without *B. cinerea* infection.

To unravel the molecular mechanisms of DN16-mediated ISR in cucumber plants, DEGs were identified by comparing analyses of the control and inoculated plants, which were markedly induced by DN16. Herein, 5-week-old cucumber plants cultivated in the sterile soils were treated with DN16 for 5 days, a total of 933 DEGs were screened, including 284 downregulated and 649 upregulated DEGs (Supplementary Table S2). KEGG pathway enrichment analysis revealed a total of 20 enriched pathways, involving metabolic pathways, biosynthesis of secondary metabolites, phenylpropanoid biosynthesis, and other pathways (Figure 2A). GO enrichment analysis for these DEGs was categorized into biological process, cell component, and molecular function (Figure 2B). The enriched DEGs were assigned to multiple processes, such as metabolic processes, cellular process, and biological regulation, and the majority of the enriched DEGs belonged to the metabolic process.

After 5 days of inoculation, the cucumber plants were subjected to foliar treatment with *B. cinerea*. A total of 681 and 519 DEGs were screened at 24 (Supplementary Table S3) and 48 hpi (Supplementary Table S4), respectively, which were distinctly induced in the pathogen-infected plants colonized by DN16. As shown in Figure 2C, Venn diagram revealed that 34 upregulated DEGs were shared in the DN16-inoculated plants, which was likely related to the DN16-mediated disease resistance (Supplementary Table S5). Among these shared DEGs, the expression of a thermospermine synthase *ACL5* gene (*CsACL5*) was induced by DN16. Moreover, 54 upregulated DEGs were significantly enriched in the inoculated plants at 24 and 48 hpi. Among these DEGs, several pathogen defense-related genes including pathogenesis-related protein 1 (*CsPR1*), defensin-like protein 1 (*CsDLP1*), and PA catabolic genes including polyamine oxidase 1 (*CsPAO1*), polyamine oxidase 5 (*CsPAO5*), and copper amine oxidase 1 (*CsCuAO1*) were significantly enriched in the pathogen-infected plants colonized by DN16 (Figure 2D). qPCR analyses showed that these PA-related genes were significantly affected in the inoculated plants, which was in line with the data of RNA-Seq (Supplementary Table S6).

### Induction of *CsACL5* by DN16 Contributed to Increased Disease Resistance

**Figure 3:**
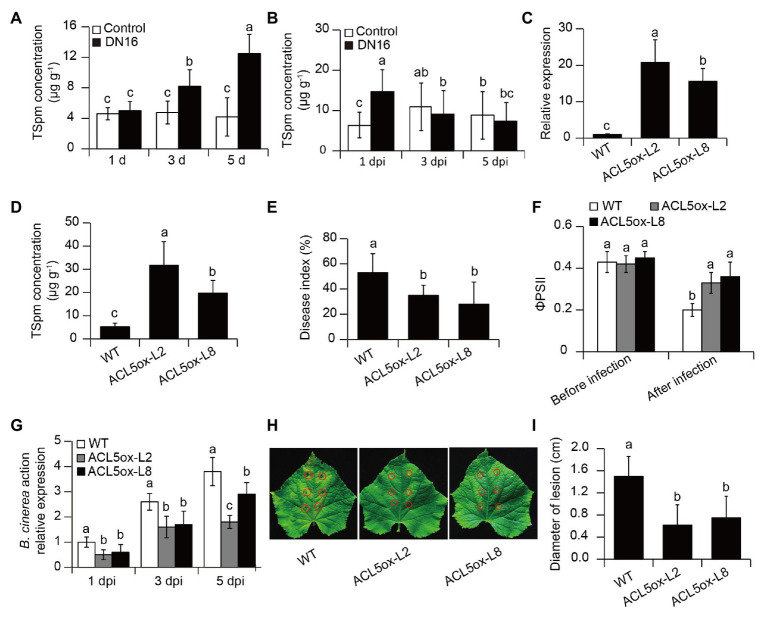
The contribution of thermospermine (TSpm) to *P. fluorescens* DN16-induced defense against *B. cinerea* infection. Five-week-old cucumber plants were treated with or without DN16, followed by *B. cinerea* infection. **(A)** After 1, 3, and 5 days of inoculation, the levels of TSpm were quantified in both the control and inoculated leaves. **(B)** The levels of TSpm in the control and inoculated leaves at 1, 3, and 5 dpi. **(C)** The expression of *CsACL5* and **(D)** leaf TSpm levels in both the wild type (WT) and two transgenic lines (ACL5ox-L2 and -L8). Five-week-old cucumber plants were treated with *B. cinerea*, and disease index **(E)**, ΦPSII **(F)**, and the expression of *BcActin* **(G)** were determined, respectively. **(H)** Lesion symptoms and **(I)** diameters of detached leaves at 5 dpi. The data represented the means ± SD from three biological repeats and different letters indicated significant differences using Duncan’s multiple range tests (*p* < 0.05).

As shown in Figure 3A, compared with the controls, the cucumber plants exhibited a marked increase of leaf TSpm levels after 3 days of DN16 inoculation. The levels of TSpm were gradually decreased in the inoculated leaves followed by the time delay of pathogen infection. After 3 days of pathogen infection, the inoculated plants displayed no marked discrepancy with the controls (Figure 3B). As mentioned above, RNA-Seq data revealed that the inoculation with DN16 induced a remarkable elevation of *CsACL5* transcripts in the cucumber leaves. To verify whether upregulation of *CsACL5* contributed to the improved host resistance against *B. cinerea*, the WT plants were transformed with *CsACL5* driven by the CaMV 35S promoter. Two independent T2 lines (ACL5ox-L2 and -L8) were used in this study, which exhibited exceedingly high expression of *CsACL5* (Figure 3C). Accordantly, the two 35S::*CsACL5* lines had higher leaf TSpm levels than the WT plants (Figure 3D). We further assessed the impacts of *CsACL5* overexpression on disease resistance. At 7 dpi, the 35S::*CsACL5* lines exhibited a notable reduction in disease index compared with the WT plants (Figure 3E). The 35S::*CsACL5* lines also displayed less photosynthetic damages than the WT plants upon exposure to pathogen infection, as reflected by higher values of ФPSII in the transgenic lines (Figure 3F). qPCR analyses revealed that the transcription of *BcActin* was markedly lower in the leaves of the 35S::*CsACL5* lines than the WT plants (Figure 3G). Consistently, in the detached leaf pathogen tests, overexpression of *CsACL5* enhanced cucumber resistance against *B. cinerea* infection, displaying smaller lesion diameters (Figures 3H,I). These findings implied that high-level transcription of *CsACL5* conferred the increased disease resistance in cucumber plants.

**Figure 4:**
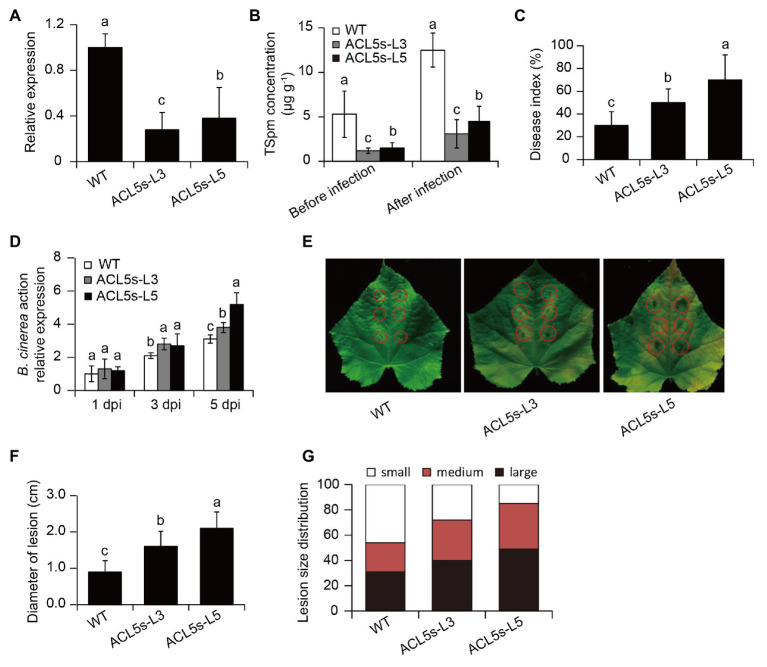
Silencing of *CsACL5* increased the susceptibility of cucumber plants to *B. cinerea* infection. **(A)** The expression of *CsACL5* in both the WT and two transgenic leaves (ACL5s-L3 and -L5). **(B)** Leaf TSpm content in both the WT and two transgenic lines before pathogen infection or after 5 days of pathogen infection. Five-week-old WT and transgenic lines were sprayed with *B. cinerea*, and **(C)** disease index and **(D)** the expression of *BcActin* were calculated at 1, 3, and 5 dpi, respectively. **(E)** Lesion symptoms, **(F)** diameters, and **(G)** size distribution of detached leaves at 5 dpi. The data represented the means ± SD from three biological repeats and different letters indicated significant differences Duncan’s multiple range tests (*p* < 0.05).

To further ascertain whether induction of *CsACL5* transcripts in cucumber plants by DN16 was responsible for the DN16-mediated disease resistance, the RNAi-*CsACL5* vector was introduced into the WT plants. qPCR analyses revealed a marked decrease in the transcription of *CsACL5* of approximate 60% in two *CsACL5*-silenced lines (ACL5s-L3 and -L5) compared with the WT plants (Figure 4A). Consistently, leaf TSpm levels were significantly reduced in the *CsACL5*-silenced lines compared with the WT plants (Figure 4B). The inoculation with DN16 was not able to considerably augment the levels of TSpm in the leaves of the *CsACL5*-silenced lines (Figure 4B). Furthermore, we examined the effects of *CsACL5* silencing on the DN16-mediated ISR. As shown in Figure 4C, silencing of *CsACL5* greatly weakened the DN16-induced cucumber resistance against *B. cinerea* infection. At 7 dpi, the *CsACL5*-silenced lines displayed the increased disease index compared with the WT plants. qPCR analyses showed that the expression of *BcActin* was distinctly less in the leaves of the *CsACL5*-silenced lines than the WT plants (Figure 4D). Accordantly, detached leaves of the *CsACL5*-silenced lines exhibited more serious necrotic tissues and larger lesion diameters than the WT plants at 5 dpi (Figures 4E,F). Additionally, the WT plants had lower proportions of fewer small lesions (lesion diameter < 5 mm) than the *CsACL5*-silenced lines (Figure 4G).

### PA Catabolism Is Essential for DN16-Induced Resistance Against *B. cinerea*

**Figure 5:**
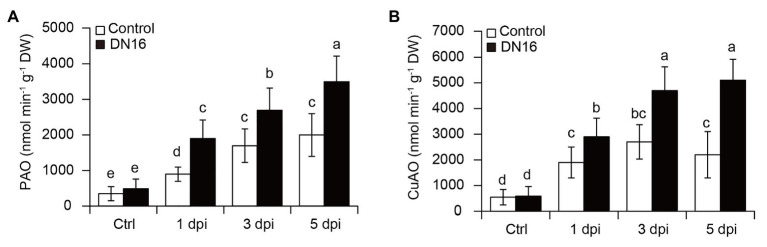
Changes of copper amine oxidase (CuAO) and polyamine oxidase (PAO) activities in both the control and DN16-inoculated leaves in response to *B. cinerea* infection. Detached leaves were treated with *B. cinerea* at the indicated times. **(A)** The activities of PAOs and **(B)** CuAOs were quantified in both the non-pathogen‐ (Ctrl) and pathogen-infected leaves in the absence or presence of DN16. The data represented the means ± SD from three biological repeats and different letters indicated significant differences using Duncan’s multiple range tests (*p* < 0.05).

Pathogen-mediated activation of defense responses is concomitant with the stimulated oxidation of cellular PAs in plants (Hatmi et al., 2014, 2015). The increased disease resistance is positively associated with the promoted PA catabolism in the pathogen-infected plants. In this study, the RNA-Seq data mentioned above showed that the transcription of PA catabolic genes (*CsPAO1*, *CsPAO5*, and *CsCuAO1*) was remarkably upregulated in the pathogen-infected leaves, but their expression was observably higher in the inoculated leaves. Furthermore, the PAO activities were measured in both the pathogen-infected control and inoculated leaves in the presence or absence of DN16. Before pathogen infection, there were no significant differences in the PAO and CuAO activities between the control and inoculated plants (Figure 5A). Upon exposure to *B. cinerea* infection, the activities of PAOs and CuAOs were progressively elevated followed by the time delay of pathogen infection. However, the inoculated plants had higher PA catabolic enzymatic activities than the controls (Figure 5B).

**Figure 6:**
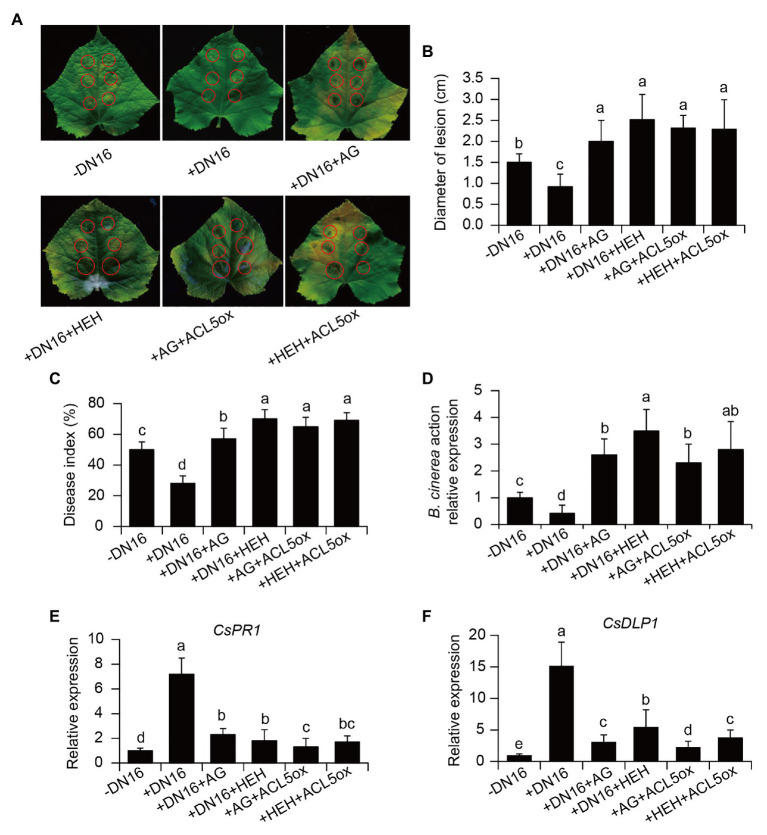
Effects of PA oxidation on the DN16-induced defense against *B. cinerea*. The control, inoculated, and *ACL5*-overexpressing plants were pre-treated with aminoguanidine (AG) or β-hydroxyehtylhydrazine (HEH) for 24 h. These plants were then subjected to *B. cinerea* infection. **(A)** Lesion symptoms and **(B)** diameters of detached leaves at 5 dpi. Disease index **(C)**, the expression of *BcActin* **(D)**, *CsPR1* **(E)**, and *CsDLP1* **(F)** were determined in cucumber plants at 7 dpi. Control, −DN16; DN16-inoculated plants, +DN16; DN16-inoculated plants treated with AG (+DN16+AG) or HEH (+DN16+HEH); *ACL5*-overexpressing plants treated with AG (+AG+ACL5ox) or HEH (+HEH+ACL5ox). The data represented the means ± SD from three biological repeats and different letters indicated significant differences using Duncan’s multiple range tests (*p* < 0.05).

We further examined whether the oxidation of TSpm was involved in the DN16-induced cucumber resistance against *B. cinerea*. For this purpose, both the control and inoculated plants were pre-treated with the PAO inhibitor HEH for 3 days, and were then infected with *B. cinerea*. The disease-resistant ability of plants was evaluated by observing the necrosis on the pathogen-infected leaves at 5 dpi. As shown in Figure 6A, the inoculation with DN16 enhanced leaf resistance against *B. cinerea* infection. The diameters of necrotic lesion were significantly smaller in the DN16-inoculated leaves than the controls (Figure 6B). However, foliar treatment with HEH resulted in the increased necrotic lesions. Accordantly, HEH treatment fully abrogated the DN16-induced disease resistance, displaying higher disease index (Figure 6C). Overexpression of *CsACL5* also failed to the increased disease resistance of cucumber plants pre-treated with HEH. Moreover, qPCR analyses showed that the transcription levels of *BcActin* were remarkably higher in the HEH-treated leaves than the control leaves. Our findings suggested a pivotal role of TSpm oxidation in mediating the defense of cucumber plants against *B. cinerea* infection.

The PAO-mediated oxidation of TSpm is associated with the production of Put, which can be further oxidized by CuAOs. This indicated that the DN16-induced resistance to *B. cinerea* was likely resulted from the CuAO-mediated Put oxidation. As shown in Figures 6A–D, the function of CuAOs in the DNA16-mediated ISR was verified by the data that the CuAO inhibitor AG abrogated the DN16-induced cucumber resistance against pathogen infection. Similarly, overexpression of *CsACL5* could not increase the resistance of cucumber plants against pathogen invasion when the plants were pre-treated with AG. In addition, pre-treatment with the AG or HEH failed to considerably elevate the transcription of defense-related genes including *CsPR1* and *CsDLP1* in both the DN16-inoculated and *CsACL5*-overexpressing plants after pathogen infection (Figures 6E,F). Therefore, these data indicated that the DN16-mediated enhancement of defense responses was closely related to the PAO‐ and CuAO-mediated oxidation of PAs.

## Discussion

The present study showed that beneficial rhizobacterial strain *P. fluorescens* DN16 remarkably stimulated the synthesis of TSpm and its catabolism with obvious ramifications for the interactions between the foliar pathogen *B. cinerea* and cucumber plants. Specifically, the DN16-inoculated plants accumulated higher leaf TSpm levels than the controls. The transcription levels of the *CsACL5* gene, encoding a putative TSpm synthase, were notably induced in the cucumber plants colonized by DN16. The increased TSpm level was positively correlated with upregulation of *CsACL5* transcripts in the leaves of the inoculated plants. Suppression of *B. cinerea* infection in the inoculated plants with higher level of TSpm implied that this tetraamine conferred the enhanced host resistance against this pathogen. This hypothesis was further favored by the increased tolerance to *B. cinerea* displayed by the *CsACL5*-overexpressing lines. Moreover, the inoculated plants also displayed higher transcription of PA catabolic genes than the controls. Repression of the PAO and CuAO activities distinctly abrogated the DN16-mediated ISR in cucumber plants, indicating that the abilities of PA catabolic enzymes to oxidize TSpm were responsible for the TSpm-mediated defense responses in the DN16-inoculated plants. Our findings were in accordance with the data of a previous study, in which the PAO-mediated TSpm catabolism participates in modifying *Arabidopsis* defense against *B. cinerea* infection (Marina et al., 2013).

Thermospermine is a major type of PAs widely present in plant kingdom known to induce defense responses in plants (Jiménez-Bremont et al., 2014). TSpm exogenously supplied can enhance the resistance of *Arabidopsis* plants against *P. viridiflava*, which is also similar to the observation for *Arabidopsis* plants ectopically expressing *AtACL5* (Marina et al., 2013). Mount evidence has indicated that genetic manipulation of *ACL5* homologous genes or the increased TSpm levels can enhance defense responses in plants (Sagor et al., 2012; Mo et al., 2015a). Silencing of *GhACL5* reduces the levels of TSpm and increases the susceptibility of plants to *V. dahlia*. In this study, it was worth noting that the transcription levels of *CsACL5* were markedly increased in the DN16-inoculated plants. Consistently, the inoculated plants accumulated more leaf TSpm content than the controls. Similarly, overexpression of *GhACL5* can increase the levels of TSpm in *Arabidopsis* plants (Mo et al., 2015a). We further verified whether the DN16-induced expression of *CsACL5* contributed to the increased disease resistance in cucumber plants. Our results showed that overexpression of *CsACL5* in cucumber plants led to higher accumulation of TSpm and stronger resistance to *B. cinerea*. Conversely, silencing of *CsACL5* abolished the DN16-induced resistance of plants to *B. cinerea* infection. Collectively, these results indicated that the expression levels of *CsACL5* were positively associated with the disease-resistant ability of cucumber plants to *B. cinerea* infection.

It has recently been indicated that overexpression of *AtACL5* lead to no obvious changes of the TSpm levels in *Arabidopsis* plants compared with the controls, but enhances disease resistance, which is primarily attributable to activation of *PAOs* that can mediate the catabolism of TSpm (Marina et al., 2013). Interestingly, upon exposure to pathogen infection, there was no marked discrepancy in the leaf TSpm levels between the control and DN16-inoculated plants challenged with *B. cinerea* in spite of higher TSpm levels in the inoculated plants without pathogen infection. This finding prompted us to speculate that the attacks of *B. cinerea* may trigger the expression of several genes involved in the PA catabolism, thereby promoting the catabolism of TSpm. The enhanced transcription of *CsPAO1*, *CsPAO5*, and *CsCuAO1* displayed by the pathogen-infected plants inoculated with DN16 implied that reduction of leaf TSpm levels in the inoculated plants were resulted from the enhanced activities of PA catabolic enzymes, probably participating in adjusting dynamic homeostasis of TSpm. Similar results were also observed in *Arabidopsis* plants, in which overexpression of *AtACL5* significantly upregulates the expression levels of several *PAO* genes including *PAO1*, *3*, and *5*, indicating that these PAOs play important roles in controlling the level of TSpm (Marina et al., 2013).

Either abiotic stress or and *B. cinerea* infection can also enhance the activities of PAOs and defense responses in grapevine leaves (Hussain et al., 2011; Hatmi et al., 2014, 2015). Yoda et al. (2009) have shown that the tobacco mosaic virus (TMV)-infected tobacco plants display the augmented PAO activities with higher production of apoplastic H_2_O_2_ through the PA oxidation-related pathways. The PA oxidation further contributes to the enhanced defense responses of tobacco plants against TMV infection. Apoplastic PA oxidation has previously been shown to participate in plant defense against microbial pathogens (Marina et al., 2008). Gonzalez et al. (2011) have demonstrated that the increased *Arabidopsis* resistance to *P. viridiflava* regulated by Spm is at least partially dependent on the activities of PAOs. In this study, the inoculation with DN16 failed to increase the resistance of cucumber plants against *B. cinerea* infection after treatment with the PAO or CuAO inhibitor, indicating that the PAO‐ and CuAO-mediated TSpm catabolism was essential for the DN16-induced resistance against *B. cinerea* infection. In fact, either beneficial rhizobacteria or pathogens can induce priming in plants, which has been shown to effectively provoke defense responses. A faster and stronger activation of defense responses frequently occurs in the primed plants after subsequent invasion of pathogens (Heil and Bostock, 2002; Van der Ent et al., 2009; Romera et al., 2019). Previously, volatile organic compounds can be released by caterpillar-infested plants for inducing priming protective effects for earlier and stronger activation of subsequent defenses rather than to induce a direct activation of defense responses (Ton et al., 2006). Our data revealed that the inoculation with DN16 did not largely impact the expression of most defense-related genes, but the induced defense responses were markedly enhanced in the inoculated plants infected by *B. cinerea*. However, the priming effects were fully abolished in the inoculated plants treated with the PAO or CuAO inhibitor. These results confirmed that the TSpm-mediated priming was a more valid defense strategy, involving the DN16-induced cucumber resistance against *B. cinerea*.

## Conclusion

**Figure 7:**
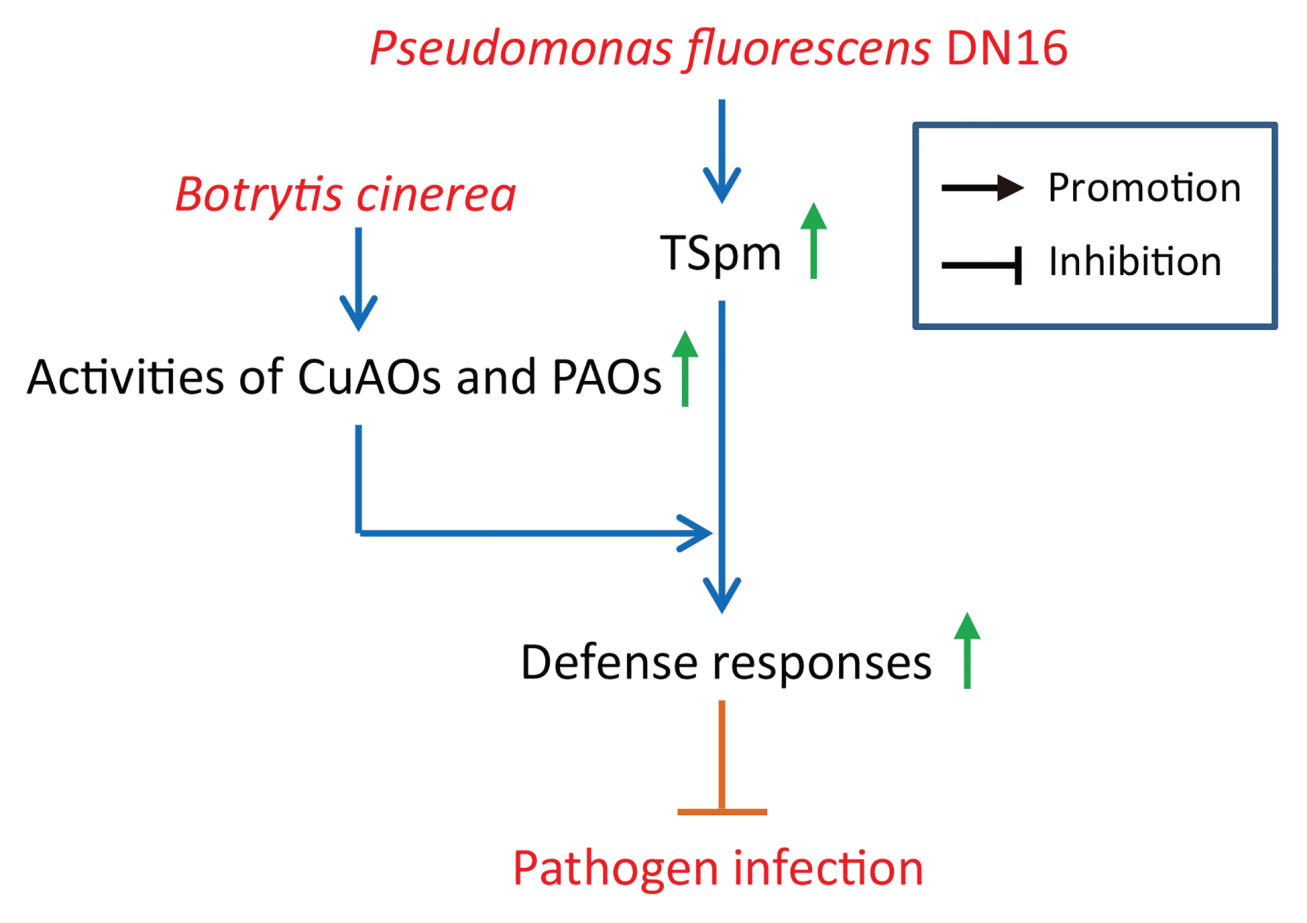
A proposed model for illustrating the functions of TSpm oxidation in the rhizobacteria-mediated cucumber resistance against *B. cinerea*. *Pseudomonas fluorescens* DN16 increased leaf TSpm levels in cucumber plants, but did not trigger the expression of pathogenesis-related genes, conferring a primed state of enhanced defense in cucumber plants. Upon exposure to *B. cinerea*, the catabolism of TSpm was quickly activated, involving the stimulation of the CuAO and PAO activities. This further contributed to the increased host resistance against pathogen infection.

Our study has unraveled an important role of beneficial rhizobacterial strains as a part of cucumber immune systems against *B. cinerea*. To our knowledge, these findings provide novel evidence for the tripartite interactions between rhizobacteria, plants, and foliar pathogen. Herein, a model was provided for the DN16-induced cucumber resistance against *B. cinerea* (Figure 7), whereby the biosynthesis of TSpm is substantially induced by DN16, resulting in higher leaf TSpm levels. Upon exposure to *B. cinerea* infection, the PAO and CuAO activities can be quickly activated to promote the catabolism of TSpm in the inoculated plants, thereby effectively provoking defense responses.

## Data Availability Statement

The datasets presented in this study can be found in online repositories. The names of the repository/repositories and accession number(s) can be found in the article/Supplementary Material.

## Author Contributions

LQ and HD: conceptualization and supervision. LZ, NQ, and YS: investigation and formal analysis. LQ and LZ: funding acquisition. LZ and NQ: experiments. LZ, NQ, and XL: analysis of results. LZ, LQ, and XL: writing original draft. HD, YS, NQ, and LZ: review and editing. All authors contributed to the article and approved the submitted version.

### Conflict of Interest

The authors declare that the research was conducted in the absence of any commercial or financial relationships that could be construed as a potential conflict of interest.

## Footnotes

^1^http://cucurbitgenomics.org
