# Supplementary material for: Pseudomonas fluorescens DN16 Enhances Cucumber Defense Responses Against the Necrotrophic Pathogen Botrytis cinerea by Regulating Thermospermine Catabolism
Source: Front Plant Sci. 2021 Feb 22;12:645338. doi: 10.3389/fpls.2021.645338 (PMC7937916; doi:10.3389/fpls.2021.645338)
Supplement: Supplementary file 1 [file Data_Sheet_1.zip › Supplementary_Material.docx]

Supplementary Material

**Table S1**. Primers used in this study.

**Table S2.** Up-regulated and down-regulated differentially expressed genes (DEGs) between the control and DN16-inoculated plants.

**Table S3.** Up-regulated and down-regulated differentially expressed genes (DEGs) between the control and DN16-inoculated plants at 24 hpi.

**Table S4.** Up-regulated and down-regulated differentially expressed genes (DEGs) between the control and DN16-inoculated plants at 48 hpi.

**Table S5.** Shared up-regulated DEGs between the control and DN16-inoculated plants.

**Table S6.** Shared up-regulated DEGs between the control and DN16-inoculated plants at 24 dpi and 48 dpi.
